# Successful Drug-Mediated Host Clearance of *Batrachochytrium salamandrivorans*

**DOI:** 10.3201/eid2902.221162

**Published:** 2023-02

**Authors:** Amadeus Plewnia, Stefan Lötters, Michael Veith, Martin Peters, Philipp Böning

**Affiliations:** Trier University, Trier, Germany (A. Plewnia, S. Lötters, M. Veith, P. Böning);; Chemisches und Veterinäruntersuchungsamt Westfalen, Arnsberg, Germany (M. Peters)

**Keywords:** fungi, Bsal, salamander plague, *Batrachochytrium salamandrivorans*, Caudata, amphibian crisis, chytridiomycosis, itraconazole, Germany

## Abstract

Skin fungi are among the most dangerous drivers of global amphibian declines, and few mitigation strategies are known. For *Batrachochytrium salamandrivorans* (Chytridiomycota), available treatments rely on temperature, partially combined with antifungal drugs. We report the clearance of *B. salamandrivorans* in 2 urodelan species using a solely drug-based approach.

Various emerging infectious diseases have been invoked in global amphibian declines and extinctions, bringing the class Amphibia as a whole to the brink of extinction (*1*). The skin-invading chytrid fungi *Batrachochytrium dendrobatidis* and *B. salamandrivorans*, both originating from Asia, are among the most noteworthy pathogens in the amphibian crisis (*1*–*3*). The few strategies that have proven to be effective in mitigating those fungi usually involve ex situ conservation breeding, which requires successful treatment protocols. Several treatments against *B. dendrobatidis* exist (*4*), based either on the pathogen’s susceptibility to high temperatures or antifungal drugs. However, antifungal drugs have been considered ineffective in mitigating the effects of *B. salamandrivorans* on its salamander hosts. Therefore, temperature-based strategies (*5*) or combinations of drug-based and temperature-based approaches seem to be the only treatment protocols for clearing *B. salamandrivorans* (*6*). Because many salamanders from temperate zones prefer low temperatures, successful treatment might be impeded by their upper thermal limit in some taxa (*7*). Moreover, those protocols require laboratory settings, which some ex situ facilities and private keepers might not have available. In addition, heat treatments are not always successful and can require repeated and prolonged application (*8*). Therefore, drug-mediated treatments can be critical in certain hosts. Furthermore, drug-based treatment protocols have been applied as viable mitigation strategies in wild amphibian populations (*9*,*10*). 

We report the successful clearance of *B. salamandrivorans* in 2 salamander species by using the antifungal drug itraconazole, commonly used in amphibian medicine (*4*,*11*,*12*). However, we stress that the potential application of our protocol requires further testing with a robust experimental setting far beyond this case.

## The Study

In early 2022, we collected 7 specimens (6 fire salamanders [*Salamandra salamandra*], specimens FS1–FS6; and 1 Alpine newt [*Ichthyosaura alpestris*], specimen AN, in terrestrial phase) demonstrating clinical symptoms of *B. salamandrivorans*–induced chytridiomycosis (circular skin lesions) at a formerly unknown outbreak site in Densborn, Eifel Mountains, Germany. Specimens were transported under strict biosecurity standards to the facilities of Trier University for subsequent *B. salamandrivorans* testing and treatment. We confirmed the presence of *B. salamandrivorans* through skin swab specimens and subsequent DNA extraction using the QIAGEN Blood and Tissue kit (QIAGEN, https://www.qiagen.com) and quantitative PCR on a StepOnePlus (ThermoFisher Scientific, https://www.thermofisher.com) following previously described protocols (*8*,*13*). Individual infection loads are expressed as DNA copies (internal transcribed spacer 1 region). We ran all samples in duplicate and set the limit of detection to 100 DNA copies (*8*). Because controlled thermal treatment could not be performed, we applied an adaptation of a previously described protocol (*4*), which has proven to be effective against *B. dendrobatidis* at our facilities. We bathed specimens in a 0.01% solution of itraconazole (Sempera Liquid 10mg/mL diluted in distilled water 1:100 to a final concentration of 100 μg/mL) for 10 minutes daily for >11 consecutive days. Individual treatment ended when the specimen tested negative. During treatment, specimens were housed individually in 20 cm × 35 cm plastic containers on moist paper towels in a climate chamber at a constant temperature of 15°C and 80% humidity; they were transferred daily after treatment to containers disinfected with Virkon S at 5g/L (https://virkon.us) and thoroughly rinsed. Animals were handled with nitrile gloves and fed crickets ad libitum. We collected skin swab specimens daily before bathing and on individual schemes for >8 weeks after treatment to check for reinfection with or regrowth of *B. salamandrivorans* (Appendix Table).

**Figure 1:**
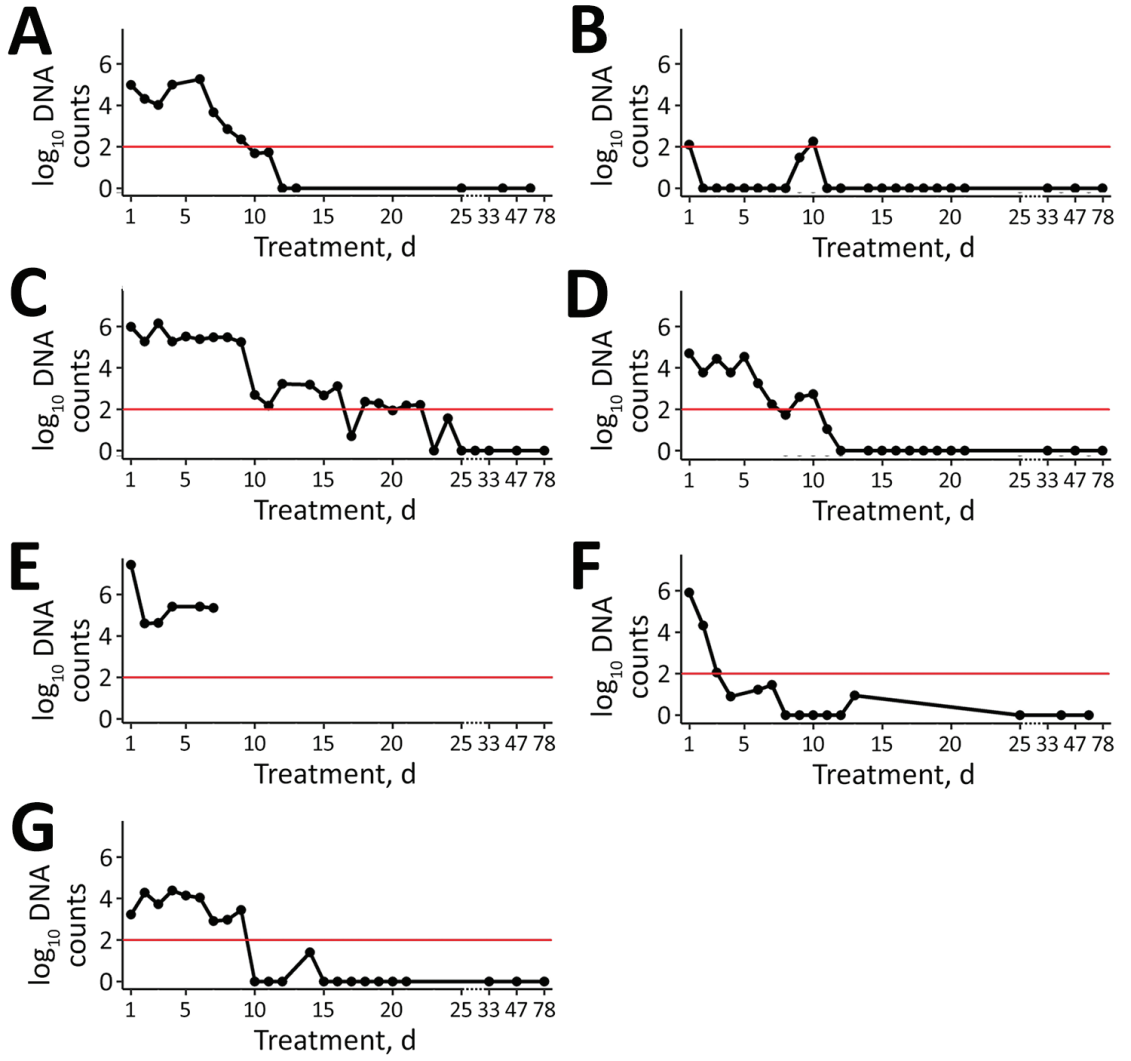
Infection loads of *Batrachochytrium salamandrivorans* in 2 urodelan species during treatment with the antifungal drug itraconazole over time. log_10_ DNA counts indicate logarithmic scale for the amount of copies of the rRNA internal spacer 1. A) Alpine newt (*Ichthyosaura alpestris*) specimen; B–G) fire salamander (*Salamandra salamandra*): B) specimen FS1; C) specimen FS2; D) specimen FS3; E) specimen FS4; F) specimen FS5; G) specimen FS6. Red line indicates limit of detection.

**Figure 2:**
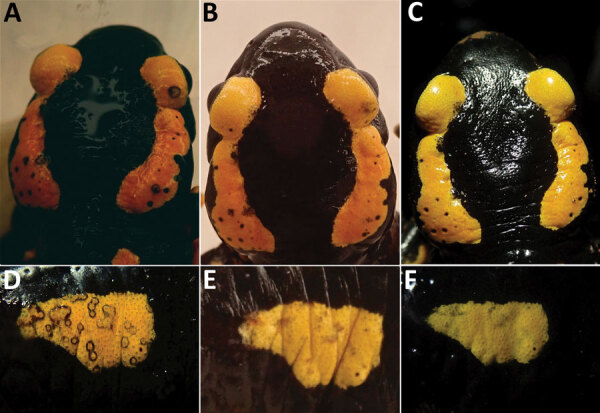
Clearing of skin lesions during the treatment of *Batrachochytrium salamandrivorans* in 2 fire salamanders (*Salamandra salamandra*) specimens, FS6 (A–C) and FS2 (D–F), on day 1 (A, D), day 16 (B, E) and day 87 (C, F).

In total, 4 specimens (AN, FS2, FS5, and FS6) demonstrated initial infection loads of 10,000–1 million DNA copies; 1 (FS4) had loads of >1 million and 2 (FS1, FS3) had loads of <100,000 (Figure 1; Appendix Table). After an initial decrease in infection loads, FS4 died on treatment day 6. We preserved FS4 in 4% buffered formalin and conducted a complete necropsy and histologic examination of skin and internal organs with regard to potential effects of treatment and severity of *B. salamandrivorans* infection and to exclude comorbidities (Appendix). In all other specimens except FS6, the infection load decreased over time. In FS6, the number of DNA copies increased over 4 days (23,972 DNA copies) and decreased thereafter (Figure 1; Appendix Table). However, we did not observe any visible progression of clinical symptoms (i.e., skin lesions) (Figure 2). After day 11, all animals except FS2 tested negative through the end of the treatment; FS2 only tested negative on day 23 (Figure 1; Appendix Table). In FS2, the infection load varied throughout treatment, ranging from 0 to 1,411,911 DNA copies (Figure 1; Appendix Table). Infection loads remained high for the first 9 days of treatment in this specimen but then steadily decreased. Initial infection loads and severe macroscopic lesions in FS2 resembled those reported in dying salamanders (*8*), suggesting an advanced stage of infection (Figure 1; Appendix Table). We observed intense skin shedding and complete healing of skin lesions throughout treatment in all specimens (Figure 2). Histopathologic examination of FS4 revealed multifocal extensive necrotic and ulcerative lesions with intralesional chytrid thalli (Appendix Figure, panel A). Furthermore, we identified multifocal secondary epidermal invasion with other unidentified fungal hyphae and bacteria (Appendix Figure, panels B, and C). Neither in the epidermis nor the liver (Appendix Figure, panel D) or other organs (not shown) were lesions present that clearly indicate toxic effects of the treatment.

## Conclusions

Blooi et al. (*5*) demonstrated a lower MIC of itraconazole for *B. salamandrivorans* than for *B. dendrobatidis*. However, they were unsuccessful in clearing *B. salamandrivorans* infections with itraconazole. Their protocol differed from ours in concentration (0.6 μg/mL), mode of application (spraying), duration (application 2×/d for 10 d) and husbandry conditions (no daily transfer to disinfected cages). Itraconazole has also been used for in situ mitigation of *B. dendrobatidis*, which increased population survival in the midterm (*9*,*10*,*12*). The exceptionally high levels of DNA copies we detected in some specimens along with severe skin lesions during the first days of treatment demonstrate that itraconazole is capable of effectively clearing even high infection loads of chytridiomycosis caused by *B. salamandrivorans* in *S. salamandra* salamanders (*2*, *8*) (Figures 1, 2). The high variation in infection intensity over consecutive days might be because of excessive skin shedding, a common response of amphibians to chytrid fungi (*14*). Moreover, we cannot rule out the possibility that handling specimens with nitrile gloves affected our results, because their runoff impairs the viability of *B. salamandrivorans* zoospores (*15*). However, whether nitrile glove runoff contributes to *B. salamandrivorans* clearance on live specimens is unknown. Furthermore, our applied husbandry conditions could have positively affected the rapid clearance of the salamander fungus. In other studies (*4*–*6*,*8*), only moist tissues were changed, possibly leaving zoospores in the enclosure that could promote reinfection. During and after treatment, we observed no negative side effects such as those discussed in other studies using itraconazole in amphibians (*4*,*11*). The death of specimen FS4 was most likely caused by the severity of multifocal necroulcerative skin lesions with intralesional *B. salamandrivorans* and secondary fungal and bacterial infection and not as a direct result of itraconazole toxicity (Appendix Figure, panels A–C). The successful healing of multiple and severe skin lesions in the other specimens is consistent with the findings and observations of Schulz et al. (*8*), suggesting itraconazole could be an effective future addition to currently available treatments. However, our treatment primarily intended on-hand curation of infected urodelans and therefore lacks a thorough experimental design, such as negative and positive controls. Therefore, this study must be seen as a case report, strictly requiring additional investigation before applying our protocol as a regular treatment option. Nevertheless, our preliminary findings contribute to mitigating the salamander plague and promote future studies with more robust experimental settings and in other species.

Appendix Additional information about successful drug-mediated host clearance of *Batrachochytrium salamandrivorans*.

## References

[1] Martel A, Blooi M, Adriaensen C, Van Rooij P, Beukema W, Fisher MC, et al. Wildlife disease. Recent introduction of a chytrid fungus endangers Western Palearctic salamanders. Science. 2014;346:630–1. 10.1126/science.1258268 25359973 PMC5769814

[2] Scheele BC, Pasmans F, Skerratt LF, Berger L, Martel A, Beukema W, et al. Amphibian fungal panzootic causes catastrophic and ongoing loss of biodiversity. Science. 2019;363:1459–63. 10.1126/science.aav0379 30923224

[3] Stegen G, Pasmans F, Schmidt BR, Rouffaer LO, Van Praet S, Schaub M, et al. Drivers of salamander extirpation mediated by *Batrachochytrium salamandrivorans.* Nature. 2017;544:353–6. 10.1038/nature22059 28425998

[4] Brannelly LA, Richards-Zawacki CL, Pessier AP. Clinical trials with itraconazole as a treatment for chytrid fungal infections in amphibians. Dis Aquat Organ. 2012;101:95–104. 10.3354/dao02521 23135136

[5] Blooi M, Martel A, Haesebrouck F, Vercammen F, Bonte D, Pasmans F. Treatment of urodelans based on temperature dependent infection dynamics of *Batrachochytrium salamandrivorans.* Sci Rep. 2015;5:8037. 10.1038/srep08037 25623498 PMC5389025

[6] Blooi M, Pasmans F, Rouffaer L, Haesebrouck F, Vercammen F, Martel A. Successful treatment of *Batrachochytrium salamandrivorans* infections in salamanders requires synergy between voriconazole, polymyxin E and temperature. Sci Rep. 2015;5:11788. 10.1038/srep11788 26123899 PMC4485233

[7] Li Z, Martel A, Bogaerts S, Göçmen B, Pafilis P, Lymberakis P, et al. Landscape connectivity limits the predicted impact of fungal pathogen invasion. J Fungi (Basel). 2020;6:205. 10.3390/jof6040205 33022972 PMC7712934

[8] Schulz V, Schulz A, Klamke M, Preissler K, Sabino-Pinto J, Müsken M, et al. *Batrachochytrium salamandrivorans* in the Ruhr District, Germany: history, distribution, decline dynamics and disease symptoms of the salamander plague. Salamandra (Frankf). 2020;56:189–214.

[9] Bosch J, Sanchez-Tomé E, Fernández-Loras A, Oliver JA, Fisher MC, Garner TW. Successful elimination of a lethal wildlife infectious disease in nature. Biol Lett. 2015;11:20150874. 10.1098/rsbl.2015.0874 26582843 PMC4685552

[10] Cook K, Pope K, Cummings A, Piovia-Scott J. *In situ* treatment of juvenile frogs for disease can reverse population declines. Conserv Sci Pract. 2022;4:e12762. 10.1111/csp2.12762

[11] Woodhams DC, Geiger CC, Reinert LK, Rollins-Smith LA, Lam B, Harris RN, et al. Treatment of amphibians infected with chytrid fungus: learning from failed trials with itraconazole, antimicrobial peptides, bacteria, and heat therapy. Dis Aquat Organ. 2012;98:11–25. 10.3354/dao02429 22422126

[12] Knapp RA, Joseph MB, Smith TC, Hegeman EE, Vredenburg VT, Erdman JE Jr, et al. Effectiveness of antifungal treatments during chytridiomycosis epizootics in populations of an endangered frog. PeerJ. 2022;10:e12712. 10.7717/peerj.12712 35036095 PMC8742549

[13] Standish I, Leis E, Schmitz N, Credico J, Erickson S, Bailey J, et al. Optimizing, validating, and field testing a multiplex qPCR for the detection of amphibian pathogens. Dis Aquat Organ. 2018;129:1–13. 10.3354/dao03230 29916388

[14] Ohmer MEB, Cramp RLC, White CR, Franklin CE. Skin sloughing rate increases with chytrid fungus infection load in a susceptible amphibian. Funct Ecol. 2015;29:674–82. 10.1111/1365-2435.12370

[15] Thomas V, Van Rooij P, Meerpoel C, Stegen G, Wauters J, Vanhaecke L, et al. Instant killing of pathogenic chytrid fungi by disposable nitrile gloves prevents disease transmission between amphibians. PLoS One. 2020;15:e0241048. 10.1371/journal.pone.0241048 33119670 PMC7595420

