# Supplementary material for: Successful Drug-Mediated Host Clearance of Batrachochytrium salamandrivorans
Source: Emerg Infect Dis. 2023 Feb;29(2):411–4. doi: 10.3201/eid2902.221162 (PMC9881767; doi:10.3201/eid2902.221162)
Supplement: Appendix — Additional information about successful drug-mediated host clearance of Batrachochytrium salamandrivorans. [file 22-1162-Techapp-s1.pdf]

Article DOI: https://doi.org/10.3201/eid2902.221162

Successful Drug-Mediated Host Clearance of *Batrachochytrium salamandrivorans*

Appendix

Appendix Table. Overview of all specimens included in study of drug-mediated host clearance of *Batrachochytrium salamandrivorans**

Specimen*	Day	Quantity (DNA copies)	Quantity standard deviation (SD)	DNA copies Log_{10}
AN	1	**97,814.55**	2,888.88	4.99
AN	2	**20,610.43**	993.33	4.31
AN	3	**10,357.56**	3.84	4.02
AN	4	**100,870.28**	1,392.31	5
AN	6	**182,388.38**	12,702.08	5.26
AN	7	**4,710.16**	30.85	3.67
AN	8	**702.09**	38.00	2.85
AN	9	**229.70**	8.66	2.36
AN	10	48.51	7.98	1.69
AN	11	54.42	12.80	1.73
AN	12	0	0	0
AN	13	0	0	0
AN	25	0	0	0
AN	39	0	0	0
AN	70	0	0	0
FS1	1	**124.99**	50.02	2.1
FS1	2	0	0	0
FS1	3	0	0	0
FS1	4	0	0	0
FS1	5	0	0	0
FS1	6	0	0	0
FS1	7	0	0	0
FS1	8	0	0	0
FS1	9	30.18	6.94	1.48
FS1	10	**181.96**	16.19	2.26
FS1	11	0	0	0
FS1	12	0	0	0
FS1	14	0	0	0
FS1	15	0	0	0
FS1	16	0	0	0
FS1	17	0	0	0
FS1	18	0	0	0
FS1	19	0	0	0
FS1	20	0	0	0
FS1	21	0	0	0
FS1	33	0	0	0
FS1	47	0	0	0
FS1	78	0	0	0
FS2	1	**961,044.81**	13,775.94	5.98
FS2	2	**187,159.45**	2,379.12	5.27
FS2	3	**1,411,910.87**	731,212.19	6.15
FS2	4	**187,675.69**	25,583.81	5.27
FS2	5	**323,325.87**	10,057.46	5.51
FS2	6	**247,256.05**	13,091.20	5.39
FS2	7	**302,326.41**	10,131.12	5.48
FS2	8	**305,274.94**	10,105.11	5.48
FS2	9	**172,268.50**	1,103.88	5.24
FS2	10	**501.96**	0.99	2.7
FS2	11	**146.93**	8.56	2.17
FS2	12	**1,703.93**	126.53	3.23
FS2	14	**1,558.95**	22.21	3.19
FS2	15	**470.53**	31.41	2.67

Specimen*	Day	Quantity (DNA copies)	Quantity standard deviation (SD)	DNA copies Log_{10}
FS2	16	**1,304.81**	7.57	3.12
FS2	17	5.00	3.00	0.7
FS2	18	**236.67**	37.24	2.37
FS2	19	**196.57**	1.03	2.29
FS2	20	89.10	7.14	1.95
FS2	21	**155.96**	52.93	2.19
FS2	22	**162.45**	21.46	2.21
FS2	23	0	0	0
FS2	24	36.27	28.59	1.56
FS2	25	0	0	0
FS2	26	0	0	0
FS2	33	0	0	0
FS2	47	0	0	0
FS2	78	0	0	0
FS3	1	**50,512.43**	317.95	4.7
FS3	2	**6,078.08**	46.76	3.78
FS3	3	**27,689.05**	394.25	4.44
FS3	4	**5,954.38**	1,185.35	3.77
FS3	5	**34,195.77**	272.26	4.53
FS3	6	**1,823.10**	98.90	3.26
FS3	7	**173.51**	39.94	2.24
FS3	8	54.32	4.12	1.73
FS3	9	**398.87**	27.27	2.6
FS3	10	**540.63**	67.15	2.73
FS3	11	11.15	4.51	1.04
FS3	12	0	0	0
FS3	14	0	0	0
FS3	15	0	0	0
FS3	16	0	0	0
FS3	17	0	0	0
FS3	18	0	0	0
FS3	19	0	0	0
FS3	20	0	0	0
FS3	21	0	0	0
FS3	33	0	0	0
FS3	47	0	0	0
FS3	78	0	0	0
FS4	1	**27,673,266.00**	655,513.44	7.44
FS4	2	**40,631.60**	305.72	4.61
FS4	3	**42,650.40**	2,344.67	4.63
FS4	4	**263,280.81**	6,193.92	5.42
FS4	6	**261,867.19**	343.80	5.42
FS5	1	**835,023.63**	28,024.37	5.92
FS5	2	**21,582.07**	1,550.41	4.33
FS5	3	**116.21**	27.19	2.06
FS5	4	7.93	7.34	0.9
FS5	6	17.10	4.77	1.23
FS5	7	28.75	7.47	1.46
FS5	8	0	0	0
FS5	9	0	0	0
FS5	10	0	0	0
FS5	11	0	0	0
FS5	12	0	0	0
FS5	13	9.48	7.30	0.95
FS5	25	0	0	0
FS5	39	0	0	0
FS5	70	0	0	0
FS6	1	**1,692.20**	173.35	3.23
FS6	2	**19,124.52**	1,177.04	4.28
FS6	3	**5,221.63**	142.21	3.72
FS6	4	**23,972.42**	879.63	4.38
FS6	5	**14,026.67**	806.26	4.15
FS6	6	**11,001.19**	163.21	4.04
FS6	7	**820.75**	28.02	2.91
FS6	8	**944.87**	52.88	2.98
FS6	9	**2,789.34**	124.68	3.45
FS6	10	0	0	0
FS6	11	0	0	0
FS6	12	0	0	0

Specimen*	Day	Quantity (DNA copies)	Quantity standard deviation (SD)	DNA copies Log_{10}
FS6	14	25.14	6.37	1.4
FS6	15	0	0	0
FS6	16	0	0	0
FS6	17	0	0	0
FS6	18	0	0	0
FS6	19	0	0	0
FS6	20	0	0	0
FS6	21	0	0	0
FS6	33	0	0	0
FS6	47	0	0	0
FS6	78	0	0	0

*Duration of treatment per specimen are expressed in days and detected quantity of DNA copies indicated with standard deviation and Log_{10}-transformed values of the DNA copies (Figure 1, https://wwwnc.cdc.gov/EID/article/29/2/22-1162-F1.htm). DNA copies in bold define results above limit of detection. Grey cells define samples taken after end of active treatment. AN = Alpine newt (*Ichthyosaura alpestris*); FS1–6 = Fire salamander (*Salamandra Salamandra*).

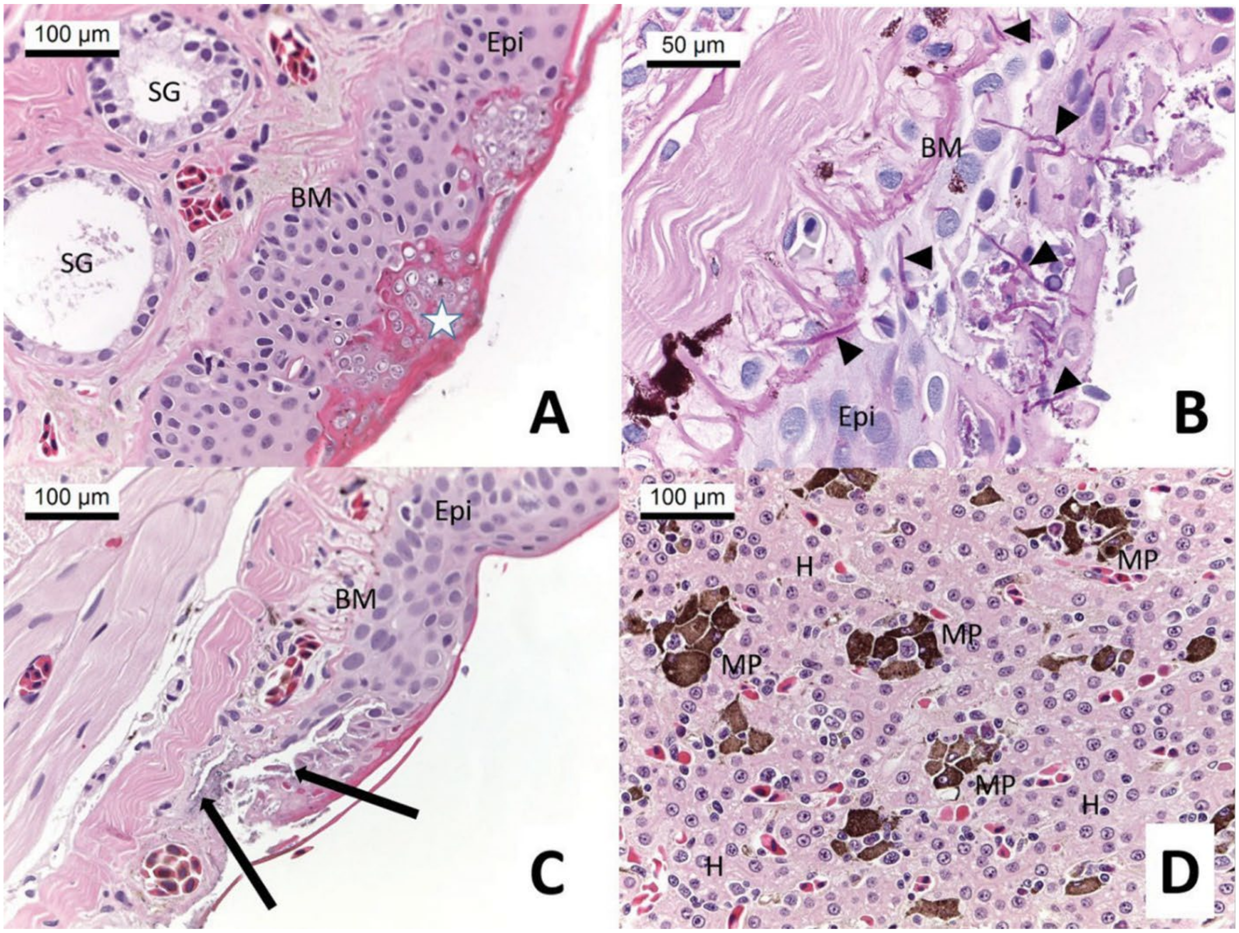

Appendix Figure. A) Focal epidermal necrosis with intralesional *Bsal* thalli (star) in specimen FS4, HE stain; B) infiltrating fungal hyphae (arrowheads) in the epidermis of specimen FS4, PAS stain; C) bacteria within ulcerative epidermal lesions (arrows) of FS4, HE stain; D) liver of FS4 with multifocal melanin pigments (MP) but without obvious toxic effects on hepatocytes (H), HE stain. BM, basal membrane; Epi, epidermis; SG, serous gland.
